# mSphere Legacy: Sharing wisdom at the culmination of a scientific career

**DOI:** 10.1128/msphere.00376-26

**Published:** 2026-07-15

**Authors:** Ira J. Blader

**Affiliations:** 1 Department of Biomedical Sciences and Pathobiology, Virginia Maryland College of Veterinary Medicine, Virginia Tech 1757, Blacksburg, Virginia, USA

## EDITORIAL

“We will be known forever by the tracks we leave.”

Dakota (Sioux) proverb

A scientist’s retirement marks the culmination of a sustained career and the beginning of new chapters and life experiences. For institutions, retirement creates opportunities to reallocate resources and elevate the next generation of scientists into new roles and leadership positions. But for those of us (yours truly included) beginning to ponder and plan for retirement, the prospect can feel daunting. It often conjures up thoughts about how our life’s work and progress will be remembered, built upon, and used. Navigating these thoughts and emotions can be challenging, particularly when retirement is framed as the end of a lifelong passion for discovery.

In conversations with colleagues, I’ve come to realize that some also associate retirement with a sense of disconnection from their community. From these conversations, I have wondered why we are unable to better emulate the tradition of many cultures in which retirement is not perceived as an end to a career but a transition to a new role that emphasizes mentorship and the sharing of hard-earned wisdom.

In today’s chaotic scientific world where each day seemingly brings new challenges, learning how past trials and tribulations were met and overcome provides a blueprint for how to stand steady in the face of these challenges. But how to share these reflections? As a society journal focused on serving the microbial sciences community, *mSphere* believes that part of its role in the scientific publishing ecosystem is to provide a place for this kind of wisdom sharing.

Thus, we are launching a new article series, mSphere Legacy, with the primary goal of providing the platform for laying the proverbial tracks for those who follow. If you are beginning to plan your retirement or even if you’ve already retired, we welcome you to share personal reflections and experiences from your career. Contributions might explore how your field has evolved since you started as a new principal investigator, how institutions responded to major scientific or societal challenges, and what excites you most about the future of the discipline.

If you are interested in contributing an mSphere Legacy commentary and would like to discuss ideas, scope, or expectations, we encourage you to reach out to me or a senior editor. An early conversation can help shape your contribution and ensure it aligns well with the goals of the series.

